# Impact of Pulsatile Bidirectional Cavopulmonary Shunt on Pre-Fontan Hemodynamics in Single Ventricle Physiology: A Meta-Analysis Reveals Favorable Outcomes

**DOI:** 10.5761/atcs.ra.24-00170

**Published:** 2025-02-27

**Authors:** Ketut Putu Yasa, I Wayan Sudarma, I Komang Adhi Parama Harta, Putu Febry Krisna Pertiwi

**Affiliations:** 1 Cardiothoracic and Vascular Surgery Division, Department of Surgery, Faculty of Medicine, Udayana University, Prof. Dr. I.G.N.G. Ngoerah General Hospital, Denpasar, Bali, Indonesia; 2 Faculty of Medicine, Udayana University, Prof. Dr. I.G.N.G Ngoerah General Hospital, Denpasar, Bali, Indonesia

**Keywords:** congenital heart disease, single ventricle physiology, bidirectional cavopulmonary shunt, bidirectional Glenn procedure

## Abstract

**Purpose:** This study aims to determine the outcomes of maintaining antegrade pulmonary blood flow (APBF) during the bidirectional cavopulmonary shunt (BCPS) procedure in patients with single ventricle physiology undergoing staged palliative surgeries.

**Methods:** A systematic search of electronic databases was conducted and focused on studies comparing pulsatile BCPS (with APBF) with non-pulsatile BCPS (without APBF). Outcomes were categorized into early (post-BCPS) and late (pre-Fontan). Data were analyzed using Mantel-Haenszel random effects model with odds ratios (ORs) and mean differences (MDs) with 95% confidence intervals (CIs). The study protocol was registered in the PROSPERO (CRD42024586369).

**Results:** A total of 17 studies with 2504 patients were included. There was no significant difference in 30-day mortality (OR 1.11, 95% CI: 0.61–2.04, p = 0.73), but pulsatile BCPS led to a higher rate of prolonged chest drainage (OR 2.45, 95% CI: 1.43–4.20, p <0.001). Pulsatile BCPS resulted in significantly higher SaO_2_ in both post-BCPS (MD 3.33%, 95% CI: 2.70–3.97, p <0.001) and pre-Fontan (MD 2.91%, 95% CI: 2.51–3.31, p <0.001). The Nakata index was also higher in the pulsatile group (MD 30.67, 95% CI: 16.68–44.65, p <0.001).

**Conclusions:** Pulsatile BCPS can optimize pre-Fontan hemodynamics by improving oxygenation and pulmonary artery development. However, the increased risk of prolonged chest drainage requires careful patient selection and monitoring.

## Introduction

Single ventricle physiology encompasses a spectrum of complex congenital heart defects characterized by the presence of a single functioning ventricle, due to hypoplasia, underdevelopment, or structural abnormalities of the other ventricle.^1)^ These defects include hypoplastic left heart syndrome (HLHS), tricuspid atresia (TA), double inlet left ventricle, unbalanced atrioventricular septal defect (AVSD), mitral atresia with normal aortic root, pulmonary atresia with intact ventricular septum, and Ebstein’s anomaly of the tricuspid valve.^2)^ In such cases, the heart is unable to sustain a normal 2-ventricle circulation, necessitating staged palliative surgical interventions. The bidirectional cavopulmonary shunt (BCPS), commonly known as the Bidirectional Glenn (BDG) procedure, is a critical intermediate step in this process, diverting systemic venous blood directly to the pulmonary arteries, thus reducing the volume load on the single ventricle.^3)^ The BCPS procedure is primarily designed to optimize hemodynamic conditions before the final stage, the Fontan procedure, which aims to separate systemic and pulmonary circulations completely.^4)^

However, the BCPS delivers less pulmonary blood flow compared to a completed Fontan or normal circulation, potentially resulting in restricted pulmonary artery (PA) growth before Fontan completion. To optimize the outcomes of BCPS, antegrade pulmonary blood flow (APBF) has been devised, aiming to increase pulmonary blood flow after the procedure. The addition of APBF has shown potential benefits, including higher oxygen saturation, improved cardiac function, prevention of arteriovenous fistulas, better PA growth, and possibly lower mortality.^5–7)^ However, despite its potential benefits, the use of APBF remains controversial. While it may offer advantages in terms of oxygenation and PA development, excessive APBF can lead to volume overload, increase atrioventricular (AV) valve regurgitation, and potentially elevate PA pressures.^6)^

This meta-analysis aims to systematically evaluate the impact of pulsatile BCPS with APBF compared to non-pulsatile BCPS without APBF in single ventricle patients. We seek to provide a comprehensive understanding of the early and late outcomes associated with each approach, focusing on hemodynamic status, PA development, and potential complications. By synthesizing available evidence, we hope to inform clinical decision-making and optimize the staged palliative approach for patients with single ventricle physiology.

## Materials and Methods

This is a systematic review and meta-analysis evaluating the impact of BCPS with APBF (pulsatile group) compared to BCPS without APBF (non-pulsatile group) in patients with single ventricle physiology. The review was conducted following the Preferred Reporting Items for Systematic Reviews and Meta-Analyses (PRISMA) guidelines.^8)^ The study protocol was registered in the PROSPERO International Prospective Register of Systematic Reviews (PROSPERO ID: CRD42024586369).

### Search strategy

A systematic search was performed on electronic databases PubMed and ScienceDirect. The search was conducted until July 2024, using the keywords “Bidirectional Cavopulmonary Shunt,” “Bidirectional Glenn,” “Single Ventricle Physiology,” and “Additional Pulmonary Blood Flow (APBF),” adjusted according to the Medical Subject Headings (MeSH) Browser. To minimize bias, cohort studies that employed propensity score matching were considered for inclusion, provided the population had already been matched.

### Inclusion and exclusion criteria

The Population, Intervention, Comparison, Outcomes (PICO) method was applied for inclusion criteria. The population included patients with single ventricle physiology undergoing BCPS or BDG procedures. The intervention group consisted of patients who underwent pulsatile BCPS (with APBF), where additional pulmonary blood flow was maintained. This is typically achieved by a native PA or through techniques such as Blalock-Taussig Shunt (BT Shunt) or PA banding (PAB). The control group included patients who underwent non-pulsatile BCPS (without APBF). The pulmonary circulation relies solely on the passive venous return from the superior vena cava (SVC) to the PA, as is typical in the classic BDG or BCPS procedure. The PA is typically ligated or surgically closed. Studies were excluded if they did not provide a clear comparison between pulsatile and non-pulsatile BCPS and involved other procedures beyond the scope of BCPS (e.g., Norwood procedure).

### Outcomes of interest

The primary outcomes in this study were categorized into early and late outcomes based on the timing of assessment. Early outcomes assessed shortly after the BCPS procedure and during the immediate postoperative period: (a) 30-day mortality, defined as any death occurring within 30 days of the BCPS procedure; (b) prolonged chest drainage, defined as the need for chest tube drainage lasting more than 7 days postoperatively; (c) post-BCPS oxygen saturation levels (SaO_2_) (%); and (d) post-BCPS mean PA pressure (mPAP) (mmHg), refers to the average pressure within the PA measured after BCPS. Late outcomes are evaluated before the Fontan procedure, focusing on long-term effects of the BCPS: (a) pre-Fontan SaO_2_ levels; (b) pre-Fontan mPAP (mmHg), defined as average PA pressure measured prior to the Fontan procedure; (c) Nakata index, defined as a measure of PA growth and development, calculated as the cross-sectional area of the pulmonary arteries divided by the body surface area (in mm^2^/m^2^), used to assess the adequacy of PA development prior to Fontan completion.

### Data extraction

Data were independently extracted by three reviewers using a pre-defined data extraction form. The extracted data included study characteristics (author, year of publication, study design, sample size), population characteristics (age and preoperative hemodynamics), details of interventions, and early and late outcomes. Discrepancies between reviewers were resolved through discussion or consultation with a fourth reviewer.

### Quality assessment and statistical analysis

The risk of bias for each included study was assessed using the recommended Newcastle–Ottawa Scale (NOS) for observational studies.^9)^ Investigations were classified as having low (<5 points), moderate (5–7 points), and high quality (>7 points). A meta-analysis was performed using Review Manager 5.4.1 software. For dichotomous outcomes (e.g., 30-day mortality, prolonged chest drainage), odds ratios (ORs) with 95% confidence intervals (CIs) were calculated. For continuous outcomes (e.g., SaO_2_ levels, mPAP, and Nakata index), mean differences (MDs) with 95% CIs were used. Mantel-Haenszel with random-effects model was employed. Statistical heterogeneity was assessed using the Higgins I^2^ statistic. Publication bias was evaluated using funnel plots. A p-value <0.05 was considered statistically significant for all analyses.

## Results

**Fig. 1:**
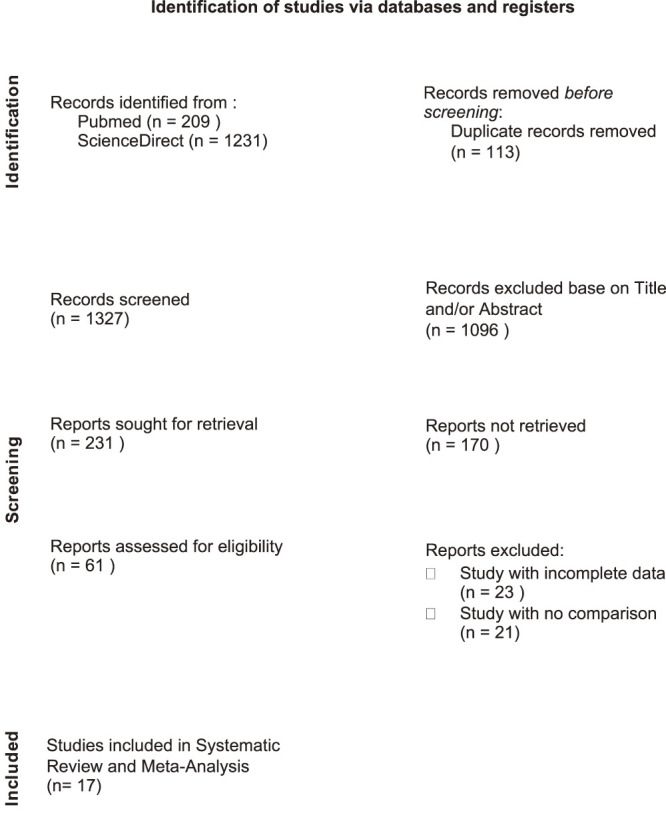
Prisma flow diagram.

The systematic search yielded a total of 1327 records. After the selection process, a total of 17 studies met the inclusion criteria and were included in this review. The selection process is illustrated in the PRISMA flow diagram (**Fig. 1**).

### Baseline characteristics

A total of 17 studies, comprising 1318 patients in the Pulsatile Group and 1186 patients in the Non-pulsatile Group, were included in this meta-analysis. The mean age of patients in the pulsatile group ranged from 5.98 to 80.3 months, while in the non-pulsatile group, it ranged from 5.52 to 73.44 months. The baseline pre-BCPS SaO_2_ levels ranged from 67.52% to 81% in the pulsatile group and from 68.19% to 80.4% in the non-pulsatile group. Various surgical techniques and interventions were employed prior to BCPS in both groups, including BT-shunt and PAB being the most performed. Predominant diagnoses also varied across studies, with TA being the most common diagnosis in several studies. The details of the baseline characteristics of the included studies are provided in **Table 1**.

**Table 1:** Baseline characteristic of included studies

Study	Group	Patients (n)	Mean age (months)	Mean SaO_2_ pre-BCPS (%)	Prior procedure (n)	Predominant diagnosis (n)
Chen, 2015^17)^	Pulsatile	57	NR	74.1	NR	NR
	Non-pulsatile	54	NR	75.2	NR	NR
Ferns, 2013^23)^	Pulsatile	33	5.98	78	NR	TA = 6
	Non-pulsatile	70	5.78	77	NR	HLHS = 28
Sughimoto, 2015^22)^	Pulsatile	60	26.4	NR	BT = 12, PAB = 12	TA = 15
	Non-pulsatile	66	14.4	NR	BT = 42, PAB = 0	PA/IVS = 17
van de Wal, 1999^7)^	Pulsatile	140	70.8	74	NR	NR
	Non-pulsatile	65	56.4	73	NR	NR
McElhinney, 1998^19)^	Pulsatile	93	NR	NR	NR	TA = 20
	Non-pulsatile	67	NR	NR	NR	HLHS = 16
Baek, 2021^15)^	Pulsatile	92	7	78	NR	NR
	Non-pulsatile	110	8	80	NR	NR
Berdat, 2005^16)^	Pulsatile	30	74.4	NR	BT = 14, PAB = 14	TA = 13
	Non-pulsatile	54	28.8	NR	BT = 35, PAB = 10	TA = 7
Caspi, 2003^14)^	Pulsatile	72	NR	NR	BT = 31, PAB = 41	Double inlet univentricular heart = 20
	Non-pulsatile	56	NR	NR	BT = 21, PAB = 0	Double inlet univentricular heart = 22
Altın, 2015^10)^	Pulsatile	87	49.4	77.6	BT = 17, PAB = 2	NR
	Non-pulsatile	34	33.9	76.7	BT = 12, PAB = 4	NR
Goel, 2001^18)^	Pulsatile	57	80.3	69.6	BT = 15, PAB = 5	TA = 42
	Non-pulsatile	38	62.5	73	BT = 15, PAB = 3	TA = 26
Nichay, 2017^20)^	Pulsatile	50	15	81	BT = 10, PAB = 10	NR
	Non-pulsatile	50	12	76	BT = 39, PAB = 14	NR
Reddy, 1997^21)^	Pulsatile	72	NR	NR	NR	NR
	Non-pulsatile	51	NR	NR	NR	NR
Yan, 2018^13)^	Pulsatile	81	73.92	67.52	NR	DORV = 11
	Non-pulsatile	31	73.44	68.19	NR	DORV = 5
Yoshida, 2005^11)^	Pulsatile	29	25.2	NR	NR	NR
	Non-pulsatile	9	22.8	NR	NR	NR
Davidson, 2023^25)^	Pulsatile	285	NR	NR	BT = 112, PAB =128	TA = 84
	Non-pulsatile	302	NR	NR	BT =71, PAB = 162	TA = 99
Gray, 2007^24)^	Pulsatile	39	NR	78	BT = 17, PAB = 8	NR
	Non-pulsatile	21	NR	78	BT = 7, PAB = 8	NR
Dietzman, 2022^12)^	Pulsatile	41	5.98	79.3	NR	NR
	Non-pulsatile	108	5.52	80.4	NR	NR

NR, not reported; BT, Blalock-Taussig shunt; PAB, pulmonary artery banding; PA/IVS, pulmonary atresia with intact ventricular septum; HLHS, hypoplastic left heart syndrome, TA, tricuspid atresia; DORV, double outlet right ventricle

In the included studies, a variety of surgical techniques were employed to maintain or eliminate APBF during the BCPS procedure. The methods for achieving pulsatile blood flow included using previous Blalock-Taussig (BT) shunts, PAB, and patent ductus arteriosus (PDA), among others. Decisions regarding whether to preserve or eliminate APBF were made intraoperatively, based on preoperative assessments, PA pressure (PAP), or systemic venous pressure. In many cases, APBF was controlled by adjusting banding techniques to maintain target mPAP. The variability in these approaches across the studies may account for differences in patient outcomes. A detailed summary of the pulsatile techniques, APBF decision-making processes, and APBF control methods across the studies is provided in **Table 2**.

**Table 2:** Summary of pulsatile techniques, APBF decision-making, and APBF control in included studies

Study	Pulsatile technique	Decision to preserve or eliminate APBF	Control of APBF
Chen, 2015^17)^	Through previous BT shunt or PA banding.	If PAP >14 mmHg, the PA is tightly banded or ligated.	Controlled, by adjusting the banding.
Ferns, 2013^23)^	PA band in patients with native pulmonary stenosis, or by tightening a previous PA band. In patients with a Sano, APBF was maintained by banding the RV to the PA shunt.	Based on preoperative measurements and subjective assessment of the decreased capacity of the pulmonary vascular bed.	Controlled. The band was adjusted until the mean Glenn pressure was around 12 torr.
Sughimoto, 2015^22)^	APF via pulmonary valve	NR	NR
van de Wal, 1999^7)^	Through the native PA, banded PA, systemic-to-pulmonary artery shunt, or PDA.	NR	NR
McElhinney, 1998^19)^	Through a banded PA, or unbanded/stenotic main PA	NR	NR
Baek, 2021^15)^	Through preexisting PBF	Do not have a clear definition regarding when to leave APBF	Controlled. The mPAP maintained no higher than 18 mmHg.
Berdat, 2005^16)^	APBF through a natively stenosed or previously banded main PA, or patent BT.	NR	NR
Caspi, 2003^14)^	Through a native stenosed pulmonary valve or a previously banded PA.	NR	Controlled. The PA band was tightened to achieve a mPAP ≤16 mmHg
Altın, 2015^10)^	From a native pulmonary valve, an adjusted antegrade PBF via PAB, and previous aortopulmonary shunts.	When the Glenn pressure was 16 mmHg, the mBT shunt was ligated, and either PAB was performed or the band was tightened.	Controlled. The PAP maintained less than 16 mmHg.
Goel, 2001^18)^	Through patent MPA	If SVC pressure >15 mmHg, the MPA is temporarily snared. If SVC pressure fell with increased SaO_2_, MPA was ligated. Other than that, MPA left the patent.	NR
Nichay, 2017^20)^	Through the pulmonary trunk or modified Blalock-Taussig shunt (MBTS).	The PA was banded to achieve target pressure in BCPS (≤16 mmHg)	Controlled, by adjusting the banding.
Reddy, 1997^21)^	Through banded or stenotic PA, or a systemic-to-pulmonary arterial shunt	NR	NR
Yan, 2017^13)^	NR	The decision to preserve the APBF or not was made intraoperatively.	NR
Yoshida, 2005^11)^	Through PA banding or previously created BT shunt	NR	Controlled. The CVP was maintained to or less than 16 mmHg immediately after BCPS.
Davidson, 2023^25)^	Through preexisting native APBF	NR	NR
Gray, 2007^24)^	Through preexisting native APBF	If PVR is believed to be low and the PAP is ≥20 mmHg, the antegrade flow is eliminated.	NR
Dietzman, 2022^12)^	Through preexisting native antegrade pulmonary blood flow	The decision to maintain APBF during this era was predominantly based on the surgeon’s preference.	NR

APBF, antegrade pulmonary blood flow; NR, not reported; PA, pulmonary artery; PDA, patent ductus arteriosus; mPAP, mean pulmonary artery pressure; PAB, pulmonary artery banding; BT, Blalock-Taussig; MPA, main pulmonary artery; SVC, superior vena cava; PVR, pulmonary vascular resistance; CVP, central venous pressure; BCPS, bidirectional cavopulmonary shunt

### Quality assessment

The quality of the included studies was assessed using the NOS. Scores ranged from 7 to 8, indicating moderate to high quality (**Supplementary Table 1**). Most studies were retrospective cohort designs, and they were assessed based on selection, comparability, and outcome domains. All included studies had adequate follow-up periods and appropriate outcome assessments.

### Early outcomes

**Fig. 2:**
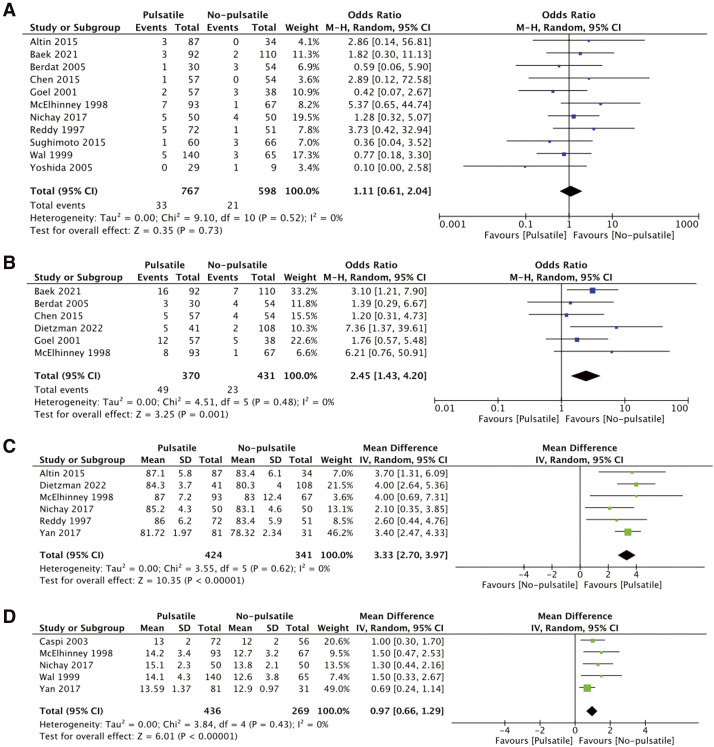
Forest plot of early outcomes. (**A**) 30-day mortality, (**B**) prolonged chest drainage, (**C**) SaO_2_ Post-BCPS, and (**D**) mPAP Post-BCPS.

There were 14 studies reporting the early outcomes.^7,10–22)^ There was no significant difference in 30-day mortality between the pulsatile and non-pulsatile BCPS groups (OR 1.11, 95% CI: 0.61–2.04, p = 0.73). This finding was consistent across studies, showing low heterogeneity (I^2^ = 0%), indicating that pulsatile BCPS does not increase short-term mortality risk. On the other hand, prolonged chest drainage was significantly more common in the pulsatile BCPS group compared to the non-pulsatile group (OR 2.45, 95% CI: 1.43–4.20, p <0.001), with low heterogeneity (I^2^ = 0%). SaO_2_ post-BCPS was significantly higher in the pulsatile group compared to the non-pulsatile group (MD 3.33 %, 95% CI: 2.70–3.97, p <0.001), with low heterogeneity (I^2^ = 0%). Additionally, the mPAP post-BCPS was slightly elevated in the pulsatile group and statistically significant (MD 0.97 mmHg, 95% CI: 0.66–1.29, p <0.001), with low heterogeneity (I^2^ = 0%). The forest plot of early outcomes is summarized in **Fig. 2**.

### Late outcomes

There were seven studies reporting the late outcomes.^11,15,17,22–25)^ Patients in the pulsatile BCPS group showed higher SaO_2_ levels pre-Fontan compared to the non-pulsatile group (MD 2.91%, 95% CI: 2.51–3.31, p <0.001), with low heterogeneity (I^2^ = 6%). No significant difference in mPAP was observed between the two groups pre-Fontan (MD 1.81 mmHg, 95% CI: –0.56 to 4.18, p = 0.13), although substantial heterogeneity was present (I^2^ = 98.0%). Additionally, the Nakata index, a measure of PA development, was significantly higher in the pulsatile BCPS group (MD 30.67, 95% CI: 16.68–44.65, p <0.001), reflecting improved PA growth, despite moderate heterogeneity (I^2^ = 60%).

**Fig. 3:**
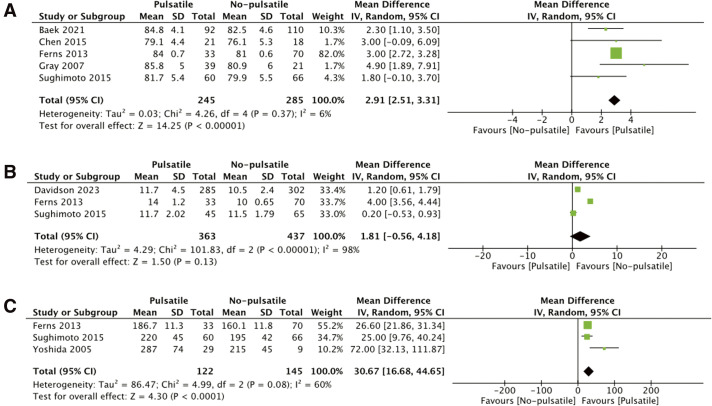
Forest plot of late outcomes. (**A**) SaO2 Pre-Fontan, (**B**) mPAP Pre-Fontan, and (**C**) Nakata Index.

The forest plot of late outcomes is summarized in **Fig. 3**.

### Publication bias assessment

Funnel plot analyses were performed for each outcome to assess publication bias and heterogeneity. For early outcomes, for example, 30-day mortality, prolonged chest drainage, mPAP post-BCPS, and SaO_2_ post-BCPS, the funnel plots were largely symmetrical, indicating minimal publication bias and consistent findings across studies. In late outcomes, the funnel plot of SaO_2_ pre-Fontan showed symmetrically. However, the other outcomes including the mPAP pre-Fontan and Nakata index funnel plots revealed substantial heterogeneity, suggesting variability across studies. While early outcomes generally showed a low risk of bias, some late outcomes exhibited biases that necessitate careful interpretation of the findings. Publication bias was provided in **Supplementary Figs. 1–7**.

## Discussion

This study represents the first meta-analysis specifically examining the outcomes of pulsatile BCPS in patients with single ventricle physiology. Our analysis demonstrated that pulsatile BCPS is associated with significantly improved oxygenation levels both immediately post-BCPS and pre-Fontan. The higher SaO_2_ levels observed in the pulsatile group highlight the potential of APBF to enhance oxygen delivery, which is crucial for the pre-Fontan stage. Furthermore, the significant increase in the Nakata index suggests that APBF promotes PA development, improving hemodynamic conditions necessary for successful Fontan completion. Despite these benefits, the addition of APBF is not without risks. The results revealed a higher incidence of prolonged chest drainage in the pulsatile BCPS. However, 30-day mortality did not differ significantly between groups, suggesting that while prolonged chest drainage is more frequent, early survival remains unaffected.

The analysis of mPAP post-BCPS showed a slight increase in the pulsatile group (MD 0.97 mmHg). This suggests that while APBF may increase pulmonary blood flow, it does not significantly elevate mPAP immediately after the procedure. The minimal heterogeneity (I^2^ = 0%) in this outcome indicates consistency across studies, supporting the notion that pulsatile BCPS can achieve enhanced flow without excessive increases in PA pressure. This is largely due to the fact that most of the APBF techniques used in these studies involved some form of APBF control. As seen in **Table 2** of pulsatile techniques, the majority of studies employed strategies to manage APBF through banding or adjusting PA pressure. For example, in several studies,^10,11,14)^ the PA was banded to maintain a target pressure, typically below 16 mmHg.

However, the mPAP pre-Fontan results presented a different picture. There was no statistically significant difference between the pulsatile and non-pulsatile groups (p = 0.11), but substantial heterogeneity (I^2^ = 98.0%) was observed. This variability suggests that the effect of APBF on long-term PA pressure may depend on several factors, including the surgical technique used, patient characteristics, and the duration between BCPS and the pre-Fontan assessment. The lack of a consistent trend in mPAP pre-Fontan across studies underscores the need for further investigation to determine how these factors influence long-term outcomes.

The findings of this meta-analysis are consistent with previous research showing that pulsatile BCPS can improve pulmonary hemodynamics and oxygenation.^13,15,17,23,24)^ PA growth is one of the major theoretical advantages of maintaining pulsatile blood flow through APBF. In this study, the Nakata index, a measure of PA development, was significantly higher in the pulsatile group, suggesting enhanced PA growth in patients with pulsatile blood flow. The presence of pulsatile flow appears to provide a growth stimulus to the pulmonary vasculature, which is essential for improving long-term outcomes, particularly in preparing patients for Fontan completion. The controlled management of APBF in these studies highlights the importance of balancing flow stimulation with pressure control, ensuring that the pulmonary circulation can grow and mature without experiencing damaging pressure increases. Previous studies have demonstrated similar results, further validating this association. Caspi et al. showed a significant increase in PA size in patients with controlled antegrade blood flow, without causing adverse increases in PA pressure or pulmonary vascular resistance.^14)^ This indicates that preserving APBF when carefully managed can promote pulmonary vessel growth without negatively impacting hemodynamics. Yoshida et al. also demonstrated that controlled APBF led to higher Fontan completion rates, attributing this success to the growth of pulmonary arteries stimulated by pulsatile blood flow.^11)^

While APBF promotes PA growth and improves oxygenation, it also poses risks such as ventricular volume overload, atrioventricular valve regurgitation (AVVR), elevated PAP, and increased central venous pressure (CVP) in the upper body.^26–28)^ The resulting rise in SVC pressure may contribute to prolonged pleural effusion, as reflected by the higher incidence of prolonged chest drainage observed in the pulsatile group in this study. A study by Januszewska et al. (2024) reported no significant differences in ventricular or AV valve function between APBF and no ABPF on pre-Fontan echocardiography. However, patients with APBF were more likely to undergo AV valve reconstruction during the Fontan operation, although this trend was not statistically significant.^29)^ Another study also found that maintaining native APBF is associated with a higher incidence of AV valve failure following Fontan completion. Notably, this association did not affect other long-term outcomes, suggesting that the impact of APBF on Fontan’s success may be limited to specific factors such as AV valve function.^25)^

Despite ongoing controversies, evidence indicates that BCPS with an “appropriate” level of controlled APBF can achieve adequate oxygen saturation without compromising ventricular function or AV valve function. In certain cases, controlled APBF during BCPS may even provide long-term palliation, particularly in patients considered high-risk for Fontan completion.^4)^ Yoshida et al. developed a surgical approach to address these issues by adjusting flow (controlled APBF) through banding the pulmonary trunk or a previously created Blalock-Taussig shunt to maintain CVP at or below 16 mmHg at the time of BCPS. This approach resulted in significantly better Fontan completion outcomes compared to cases with uncontrolled APBF.^11)^ Another study supports this finding, showing that controlled APBF by maintaining SVC pressure at or below 16 mmHg during BCPS can extend the interval between the BCPS and total cavopulmonary connection, facilitating PA growth essential for successful Fontan circulation.^28)^ Zhang et al. compared outcomes of controlled versus uncontrolled APBF in BCPS. Controlled APBF, achieved through PA banding to maintain mPAP or CVP below 16 mmHg, resulted in no significant difference in the degree of AVVR before and after BCPS. By contrast, uncontrolled APBF led to a significant increase in AVVR severity from after BCPS to before Fontan (p = 0.01). Logistic regression identified right ventricle morphology as a risk factor for worsening valve regurgitation after BCPS (OR: 3.59, p = 0.03).^26)^ A study by Kowatari et al. (2020) also reported that controlling APBF to maintain a CVP ≤18 mmHg during BCPS resulted in well-maintained Fontan circulation, with all patients achieving New York Heart Association (NYHA) class I status at long-term follow-up. Importantly, patients with controlled APBF experienced no significant adverse events, including pulmonary overcirculation or AV valve regurgitation.^27)^ These findings highlight the importance of controlled APBF in reducing the risks of excessive ventricular loading and AVVR while preserving its benefits.

Although this meta-analysis could not directly analyze the effects of AVVR, ventricular overload, or the long-term impact of APBF after Fontan, evidence from previous studies suggests that controlled APBF does not lead to AVVR and is associated with favorable outcomes post-Fontan.^4,11,26–28)^ These studies highlight that controlled APBF can address the risks of volume overload while promoting oxygenation and PA growth. By contrast, reports of increased AVVR after Fontan often lack clarity on whether the APBF was controlled, suggesting that uncontrolled flow may have contributed to these adverse outcomes. Further research is needed to confirm these findings.

Clinically, these findings offer essential insights for guiding surgical decision-making in staged palliative care. For instance, the improved oxygenation and PA growth observed with APBF suggest that pulsatile BCPS may help optimize pre-Fontan hemodynamics, potentially enhancing Fontan readiness and long-term outcomes. However, the increased incidence of prolonged chest drainage highlights the importance of selective patient management to avoid complications related to venous congestion and fluid retention. Ultimately, this study underscores the need for a personalized approach when considering APBF in BCPS, enabling surgeons to balance the benefits of enhanced pulmonary blood flow with potential hemodynamic risks based on individual patient profiles. Patients likely to benefit from pulsatile BCPS include those in whom APBF can be carefully controlled to maintain mPAP or CVP at or below 16 mmHg, along with good ventricular function and a minimal degree of AVVR. Controlled APBF in this range helps patients tolerate the additional preload while achieving improved oxygenation and PA growth. High-risk Fontan candidates may also benefit from controlled APBF to optimize pre-Fontan hemodynamics. By contrast, patients with uncontrolled APBF, severe ventricular dysfunction, or significant AVVR are at greater risk of complications, such as volume overload and worsening valve dysfunction. Proper regulation of APBF is crucial to balance its potential benefits and risks. These insights set a foundation for further research aimed at refining APBF use criteria and improving overall outcomes in this complex patient population.

### Limitations

This meta-analysis has several limitations, including the observational nature of most studies, which introduces potential bias, and the heterogeneity in patient populations and surgical techniques, particularly in managing APBF. The limited data restricted our ability to perform subgroup analyses or meta-regression. Additionally, the lack of long-term follow-up data in most studies makes it difficult to assess the full impact of APBF on patient outcomes post-Fontan. Importantly, due to limited data, we did not assess AVVR, a known disadvantage of APBF, and its impact remains unproven in this meta-analysis.

## Conclusions

In summary, pulsatile BCPS with APBF enhances pre-Fontan hemodynamics by improving oxygenation and PA development. However, it also increases the risk of complications like prolonged chest drainage, highlighting the need for careful patient selection and monitoring. While the findings are promising, further research is necessary to refine patient selection criteria, assess long-term effects on mPAP pre-Fontan, and reduce variability across studies, ultimately improving outcomes for patients with single ventricle physiology.

## Declarations

### Ethics approval and consent to participate

Not applicable.

### Consent for publication

Not applicable.

### Funding

This research received no external funding.

### Data availability

The data supporting the findings of this study are available within the article and its supplementary materials.

### Author contributions

Conception and design: KPY

Analysis and interpretation: IWS and PFKP

Data collection: IWS and IKAPH

Writing the article: IKAPH and IWS

Critical revision of the article: KPY and PFKP

Final approval of the article: IWS, PFKP, KPY, and IKAPH.

### Disclosure statement

The authors declare no competing interests.

## Supplementary Material

Supplementary Figure 1 Funnel plot of the studies reporting 30-day mortality outcomes in patients undergoing pulsatile versus non-pulsatile BCPS. The plot illustrates the relationship between the standard error of the log odds ratio (SE(log[OR])) and the odds ratio (OR) across the included studies. The dotted line represents the overall effect size. The symmetric distribution of studies suggests minimal publication bias.

Supplementary Figure 2 Funnel plot of the studies reporting prolonged chest drainage outcomes in patients undergoing pulsatile versus non-pulsatile BCPS. The plot shows the relationship between the standard error of the log odds ratio (SE(log[OR])) and the odds ratio (OR) across the studies. The dotted line represents the overall effect size.

Supplementary Figure 3 Funnel plot of the studies reporting post-BCPS oxygen saturation (SaO2) outcomes in patients undergoing pulsatile versus non-pulsatile BCPS. The plot depicts the relationship between the standard error of the mean difference (SE(MD)) and the mean difference (MD) across the studies. The dotted line represents the overall mean difference.

Supplementary Figure 4 Funnel plot of the studies reporting post-BCPS mean pulmonary artery pressure (mPAP) outcomes in patients undergoing pulsatile versus non-pulsatile BCPS. The plot shows the relationship between the standard error of the mean difference (SE(MD)) and the mean difference (MD) across studies. The dotted line represents the overall mean difference.

Supplementary Figure 5 Funnel plot of the studies reporting pre-Fontan oxygen saturation (SaO2) outcomes in patients undergoing pulsatile versus non-pulsatile BCPS. The plot shows the relationship between the standard error of the mean difference (SE(MD)) and the mean difference (MD) across studies. The dotted line represents the overall mean difference.

Supplementary Figure 6 Funnel plot of the studies reporting pre-Fontan mean pulmonary artery pressure (mPAP) outcomes in patients undergoing pulsatile versus non-pulsatile BCPS. The plot displays the standard error of the mean difference (SE(MD)) plotted against the mean difference (MD). The dotted line represents the overall mean difference.

Supplementary Figure 7 Funnel plot of the studies reporting Nakata index outcomes in patients undergoing pulsatile versus non-pulsatile BCPS. The plot shows the standard error of the mean difference (SE(MD)) plotted against the mean difference (MD). The dotted line indicates the overall mean difference.

Supplementary Table The Newcastle-Ottawa Scale qualitative analysis of the included studies
