# Supplementary material for: Impact of Pulsatile Bidirectional Cavopulmonary Shunt on Pre-Fontan Hemodynamics in Single Ventricle Physiology: A Meta-Analysis Reveals Favorable Outcomes
Source: Ann Thorac Cardiovasc Surg. 2025 Feb 27;31(1):24-00170. doi: 10.5761/atcs.ra.24-00170 (PMC11873599; doi:10.5761/atcs.ra.24-00170)
Supplement: Supplementary Figure 1 [file atcs-30-1-24-00170-s01.pdf]

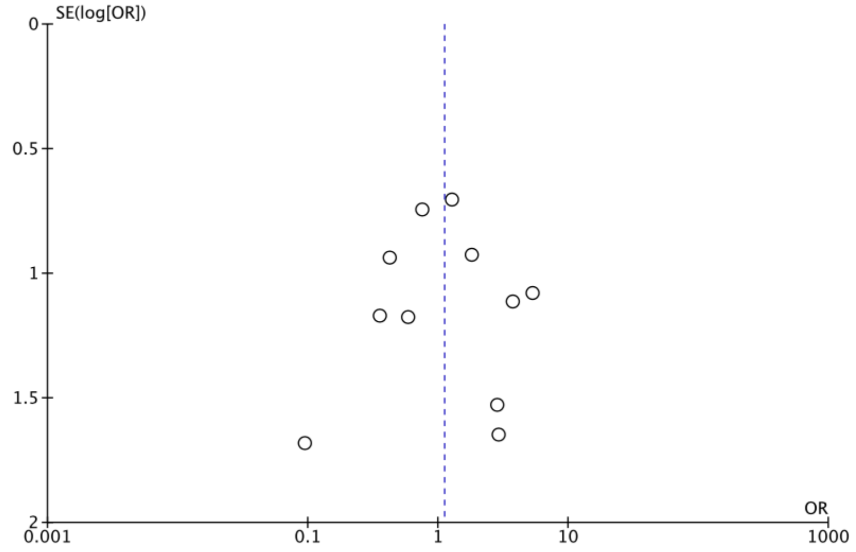

Supplementary Figure 1. Funnel plot of the studies reporting 30-day mortality outcomes in patients undergoing pulsatile versus non-pulsatile BCPS. The plot illustrates the relationship between the standard error of the log odds ratio (SE(log[OR])) and the odds ratio (OR) across the included studies. The dotted line represents the overall effect size. The symmetric distribution of studies suggests minimal publication bias.

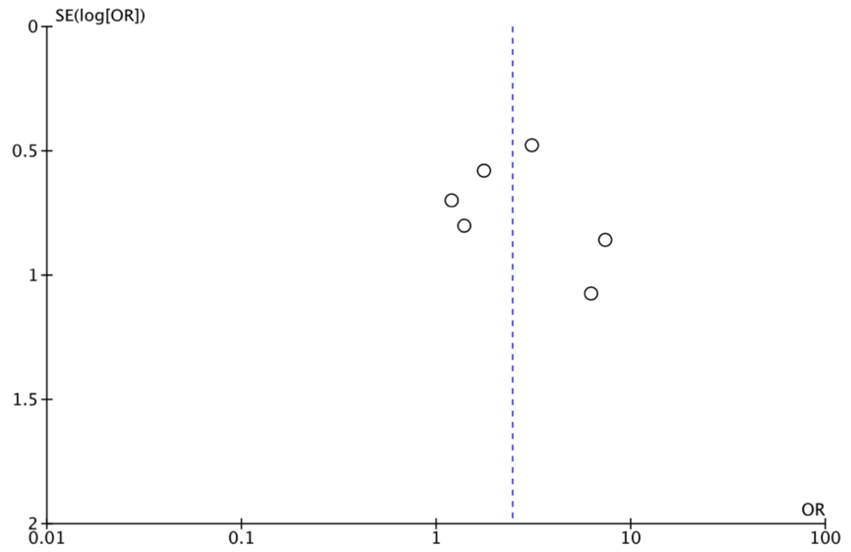

Supplementary Figure 2. Funnel plot of the studies reporting prolonged chest drainage outcomes in patients undergoing pulsatile versus non-pulsatile BCPS. The plot shows the relationship between the standard error of the log odds ratio (SE(log[OR])) and the odds ratio (OR) across the studies. The dotted line represents the overall effect size.

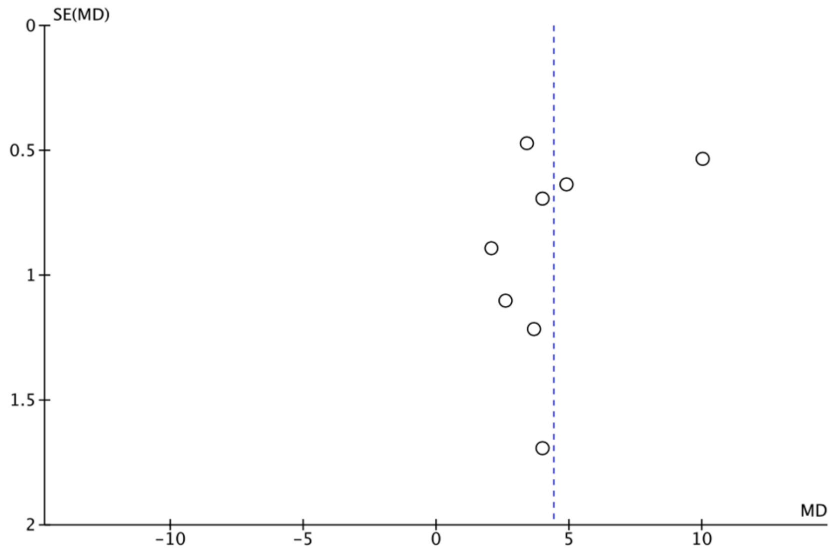

Supplementary Figure 3. Funnel plot of the studies reporting post-BCPS oxygen saturation (SaO2) outcomes in patients undergoing pulsatile versus non-pulsatile BCPS. The plot depicts the relationship between the standard error of the mean difference (SE(MD)) and the mean difference (MD) across the studies. The dotted line represents the overall mean difference.

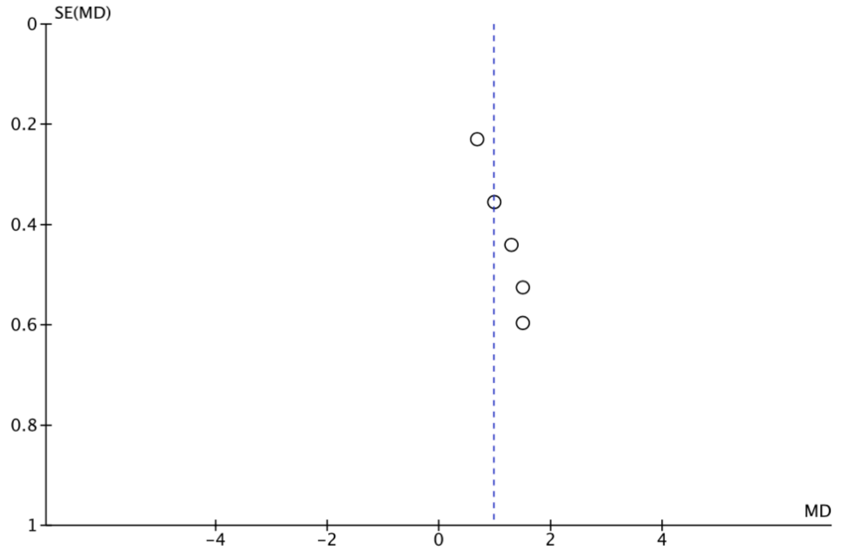

Supplementary Figure 4. Funnel plot of the studies reporting post-BCPS mean pulmonary artery pressure (mPAP) outcomes in patients undergoing pulsatile versus non-pulsatile BCPS. The plot shows the relationship between the standard error of the mean difference (SE(MD)) and the mean difference (MD) across studies. The dotted line represents the overall mean difference.

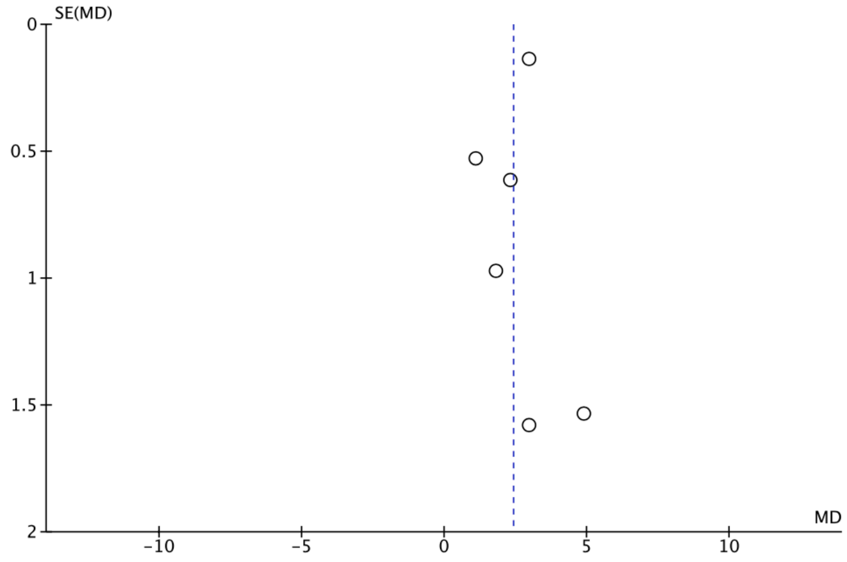

Supplementary Figure 5. Funnel plot of the studies reporting pre-Fontan oxygen saturation (SaO2) outcomes in patients undergoing pulsatile versus non-pulsatile BCPS. The plot shows the relationship between the standard error of the mean difference (SE(MD)) and the mean difference (MD) across studies. The dotted line represents the overall mean difference.

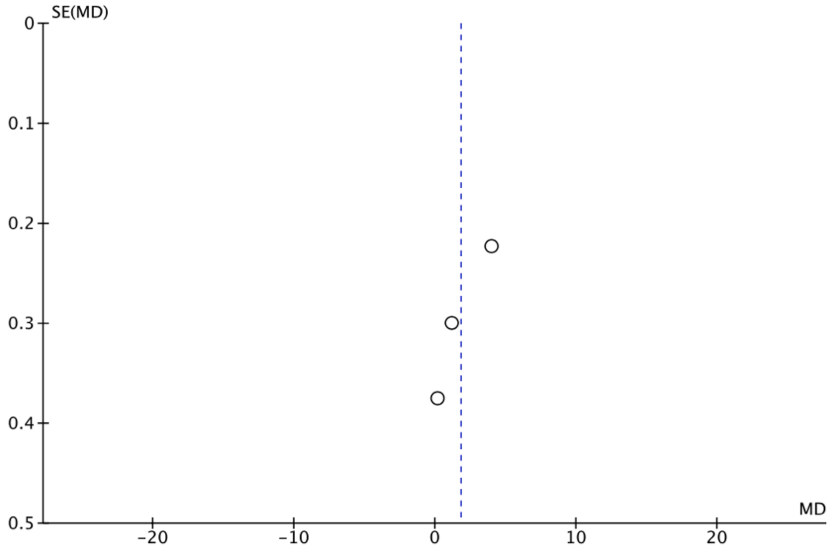

Supplementary Figure 6. Funnel plot of the studies reporting pre-Fontan mean pulmonary artery pressure (mPAP) outcomes in patients undergoing pulsatile versus non-pulsatile BCPS. The plot displays the standard error of the mean difference (SE(MD)) plotted against the mean difference (MD). The dotted line represents the overall mean difference.

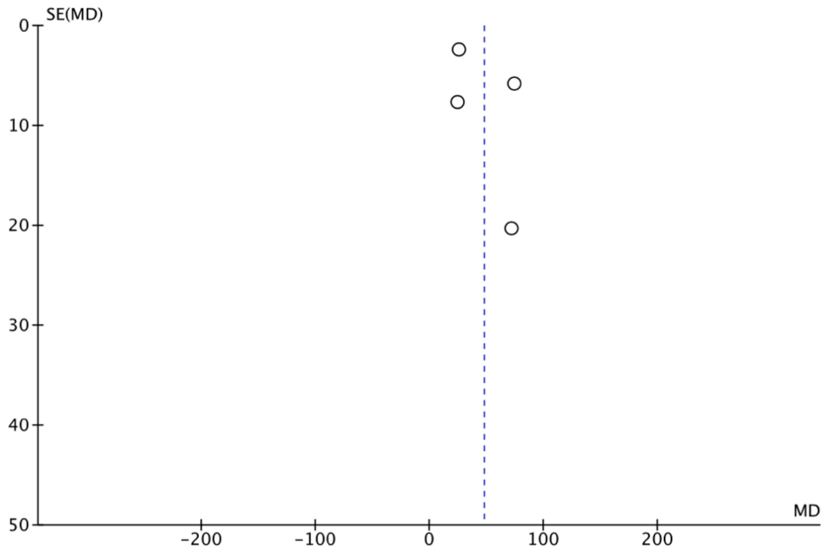

Supplementary Figure 7. Funnel plot of the studies reporting Nakata index outcomes in patients undergoing pulsatile versus non-pulsatile BCPS. The plot shows the standard error of the mean difference (SE(MD)) plotted against the mean difference (MD). The dotted line indicates the overall mean difference.
