# Supplementary material for: Impact of Pulsatile Bidirectional Cavopulmonary Shunt on Pre-Fontan Hemodynamics in Single Ventricle Physiology: A Meta-Analysis Reveals Favorable Outcomes
Source: Ann Thorac Cardiovasc Surg. 2025 Feb 27;31(1):24-00170. doi: 10.5761/atcs.ra.24-00170 (PMC11873599; doi:10.5761/atcs.ra.24-00170)
Supplement: Supplementary Table [file atcs-30-1-24-00170-s02.pdf]

Supplementary Table. The Newcastle-Ottawa Scale qualitative analysis of the included studies

Author	Study design	NOS Score			Total score
		Selection	Comparability	Outcome	
Chen 2015	Retrospective	★★★	★★	★★	7
Ferns 2013	Retrospective	★★★	★★	★★★	8
Sughimoto 2015	Retrospective	★★★	★★	★★	7
Wal 1999	Retrospective	★★★	★★	★★★	8
McElhimney 1998	Retrospective	★★★	★★	★★★	8
Baek 2021	Retrospective	★★★	★★	★★★	8
Berdat 2005	Retrospective	★★★	★★	★★★	8
Caspi 2003	Retrospective	★★★	★★	★★	7
Altin 2015	Retrospective	★★★	★★	★★★	8
Goel 2001	Retrospective	★★★	★★	★★★	8
Nichay 2017	Retrospective	★★★	★★	★★	7
Reddy 1997	Retrospective	★★★	★★	★★	7
Yan 2017	Retrospective	★★★	★★	★★	7
Yoshida 2005	Retrospective	★★★	★★	★★	7
Davidson 2023	Retrospective	★★★	★★	★★★	8
Gray 2007	Retrospective	★★★	★★	★★	7
Dietzman 2022	Retrospective	★★★	★★	★★★	8

NOS= Newcastle-Ottawa Scale
